# Basal wall hypercontraction of Takotsubo cardiomyopathy in a patient who had been diagnosed with dilated cardiomyopathy: a case report

**DOI:** 10.1186/s12872-017-0730-z

**Published:** 2017-12-12

**Authors:** Noboru Ichihara, Shuichi Fujita, Yumiko Kanzaki, Tomohiro Fujisaka, Michishige Ozeki, Nobukazu Ishizaka

**Affiliations:** Department of Cardiology, Osaka Medical College, Takatsuki-shi Daigaku-machi 2-7, Osaka, 569-8686 Japan

**Keywords:** Takotsubo cardiomyopathy, Idiopathic cardiomyopathy, Hypercontraction, Pathogenesis, Percutaneous coronary intervention, Emotional stress

## Abstract

**Background:**

Takotsubo cardiomyopathy is characterized by the basal hypercontractility and apical ballooning of the left ventriculum and T-wave inversion in the electrocardiogram. It has been suggested that Takotsubo cardiomyopathy might underlie the pathogenesis of persistent cardiac dysfunction; however, few reports are present demonstrating the advent of Takotsubo cardiomyopathy in patients with idiopathic cardiomyopathy.

**Case presentation:**

A 64-year-old women was admitted due to dyspnea on effort and lower extremity edema. She had been diagnosed with idiopathic dilated cardiomyopathy 2.5 years before owing to the reduced left ventricular ejection fraction (24%), normal coronary artery, and interstitial fibrosis of the myocardial samples. On admission, her electrocardiogram showed giant negative T wave in II, III, aVF, and precordial leads. Echocardiography showed dyskinesis of the left ventricular apex and hypercontraction of the basal wall, which had not been observed in the previous examinations. Coronary angiography showed normal coronary arteries, and apical ballooning and basal hypercontractility was confirmed by left ventriculography. On day 15 of admission, contraction of apical wall was recovered, and basal hypercontraction was disappeared.

**Conclusion:**

The present case is the first report demonstrating appearance the transient basal wall hypercontraction along with the advent of Takotsubo cardiomyopathy in a patient diagnosed with dilated cardiomyopathy. Whether such findings are indicative of fair prognosis and have the utility of understanding the pathogenesis of dilated cardiomyopathy needs further investigation.

## Background

Takotsubo cardiomyopathy is characterized by transient left ventricular apical ballooning, which typically occurs in older women after emotional or physical stress [1]. The pathophysiology of Takotsubo cardiomyopathy remains obscure, but it may occur after emotional or physical stress, so-called “triggering events”. In order to diagnose Takotsubo cardiomyopathy, several disorders that might show reversible abnormal cardiac contraction should be excluded, including obstructive coronary artery disease [2], subarachnoid hemorrhage, pheochromocytoma crisis, intracranial or subarachnoid bleeding myocarditis, tachycardia-induced cardiomyopathy, and hypertrophic cardiomyopathy [3–6]. On the other hand, the possibility exists that some of these conditions might present together with Takotsubo cardiomyopathy and underlie it as a triggering event [7, 8]. There have been few reports, until now, about Takotsubo cardiomyopathy in the dilated cardiomyopathic heart. We herein present a case of basal cardiac wall hypercontraction during the acute-phase of Takotsubo cardiomyopathy that occurred in a patient with idiopathic dilated cardiomyopathy.

## Case presentation

**Fig. 1:**
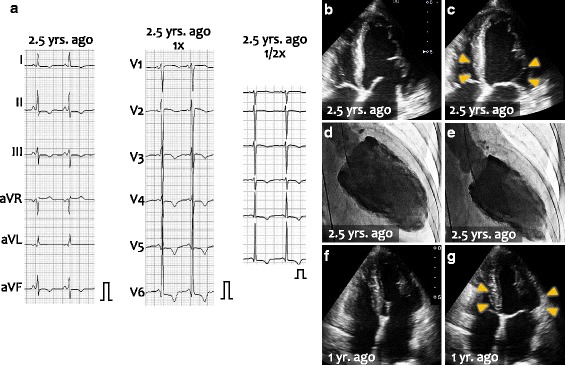
Electrocardiogram and echocardiographic images prior to the current admission. **a** Electrocardiogram 2.5 years before the current admission. T-wave inversion was observed in II, III, aVF and precordial leads. **b, c** Echocardiography 2.5 years before the current admission at end diastole (**b**) and end systole (**c**). Left ventricular wall motion was diffusely decreased including the base of the left ventricle (arrowheads).**d, e** Left ventriculogram at end diastole (**d**) and at end systole (**e**). **f, g** Echocardiography 2.5 years before the current admission at end diastole (**f**) and end systole (**g**). Wall motion of left ventricle, including the base (arrowheads) remained impaired. The calibration of the electrocardiogram indicates 1 mV

A 64-year-old woman who complained of worsening nocturnal dyspnea was admitted to our hospital. Two and half years previously, the patient had felt exertional chest pain and lower extremity edema and had been admitted to our hospital. She did not have a history of hypertension, diabetes, or smoking. On her previous admission, chest X-ray showed an enlarged cardiac silhouette with a cardiothoracic ratio of 68.7% and right side pleural effusion. Electrocardiogram showed T-wave inversion in leads II, III, aVF, and precordial leads (Fig. 1a). Echocardiography showed that left ventricular wall motion was diffusely reduced with the left ventricular ejection fraction of 24% (Fig. 1b, c). The end-diastolic left ventricular dimension was 53 mm. Coronary artery angiography showed no physiologically significant stenosis and left ventriculography showed diffuse hypokinesis of the left ventricle (Fig. 1d, e). Histological examination of endomyocardial biopsy samples showed interstitial fibrosis, but neither amyloid deposition nor granulomatous degeneration was observed. Echocardiography 1.5 years later also showed the reduced global left ventricular contractility (Fig. 1f, g).

**Fig. 2:**
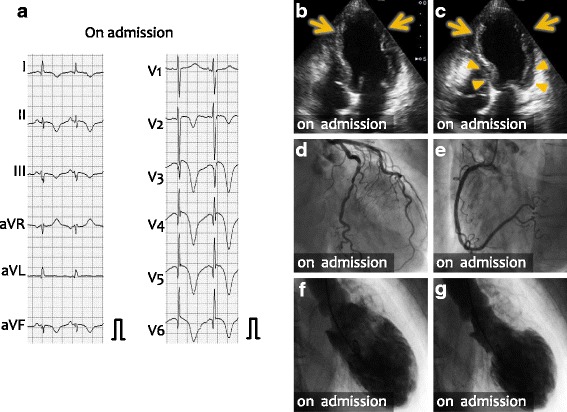
Electrocardiogram and echocardiographic and radiologic images on the current admission. **a** Electrocardiogram on the current admission. T-wave inversion became more prominent. **b, c** Echocardiography on the current admission at end diastole (**a**) and end systole (**f**). Dyskinetic wall motion was observed at the eft ventricular apex (arrows), but the base of left ventriculum showed hypercontraction. **d, e** Coronary angiography showed normal left (**d**) and right (**e**) coronary arteries. **f, g** Left ventriculogram at end diastole (**f**) and at end systole (**g**). Basal hypercontraction and apical ballooning were demonstrated

**Fig. 3:**
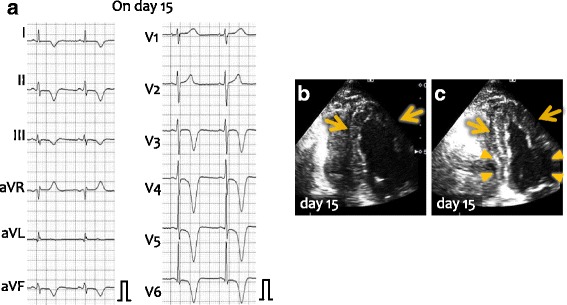
Electrocardiogram and echocardiographic images at day 15. **a** Electrocardiogram at day 15. Giant negative T waves were still present. **b, c** Echocardiography at day 15 at end diastole (**b**) and end systole (**c**). Basal hypercontraction disappeared, and thickening of the apical wall was emerging

The chief complaint of the patient on the current admission was exertional dyspnea and edema of lower extremities. On admission, her body temperature was 35.8°C, blood pressure was 104/79 mmHg, and pulse rate was 100 bpm. Electrocardiography showed T wave inversion on leads I, II, aVF, and precordial leads, which was more prominent than that observed previously (Fig. 2a). Echocardiography showed dyskinesis of the apical (Fig. 2b, c, arrows) and hypercontraction of the basal walls (Fig. 2b, c). Emergency coronary angiography showed, again, no significant stenosis in the coronary arteries (Fig. 2d, e) and apical ballooning and basal hypercontraction were demonstrated by left ventriculography (Fig. 1f, g). Laboratory examinations showed elevated levels of serum creatine kinase, its MB fraction, and plasma B-type natriuretic peptide (Table 1). In addition to the treatment with diuretic drugs, the patient was treated with anticoagulant drugs because of the thrombus formation in the left ventricular apex – intravenous administration of heparin for 12 days followed by the oral administration of warfarin. Administration of beta blocker, bisoprolol, was also started. On day 15, follow-up electrocardiogram showed no apparent changes in the giant T-wave inversion (Fig. 3a); however, echocardiography showed the disappearance of hypercontraction of the left ventricular basal wall (Fig. 3b, c, arrowheads) in addition to recovery of the contraction of the thickened apical wall (arrows). The patient was diagnosed with Takotsubo cardiomyopathy [9]. Finding of electrocardiogram, T-wave inversion, normalized at 3 months after the discharge and it remained normal at 8 months after the discharge.

**Table 1:** Laboratory data on the current admission

Blood cell count	
White blood cell count, ×10^3^/μL	11.59
Red blood cell count, ×10^6^/μL	4.93
Hemoglobin, g/dL	14.8
Platelet count, ×10^3^/μL	304
Biochemistry	
Total protein, mg/dL	8.1
serum creatinine, mg/dL	0.81
Creatine kinase, U/L	773
Creatine kinase MB, U/L	76
C-reactive protein, mg/dL	1.22
Na, mEq/L	144
K, mEq/L	4.3
Cl, mEq/L	106
BNP, pg/mL	940.8

BNP indicates brain natriuretic peptide

## Discussion

In the present case, we demonstrated the occurrence of Takotsubo cardiomyopathy in a patient who had been diagnosed with dilated cardiomyopathy. Of note, in the acute phase, the left ventricular basal wall showed hypercontraction together with the advent of apical ballooning, although these findings were transient and disappeared within 2 weeks. These findings indicated that basal wall hypercontraction can occur in patients diagnosed with dilated cardiomyopathy.

Whether there were any relationships between previously diagnosed idiopathic dilated cardiomyopathy and Takotsubo cardiomyopathy remains unclear; however, there are some possibilities. Although wall motion abnormality is, in general, transient in Takotsubo cardiomyopathy [10], several previous studies suggested that Takotsubo cardiomyopathy might be emerging as a chronic form [11], causing congestive heart failure and acute coronary syndrome-like symptoms. It is increasingly recognized that Takotsubo cardiomyopathy may not always be benign [12], and may cause left ventricular fibrosis [13] leading to appearance as a non-ischemic cardiomyopathy [14]. In addition, presence of Takotsubo cardiomyopathy may not be able to be recognized or diagnosed when it is not associated with anginal chest pain [15].

Considering that our patient had T-wave inversion in her electrocardiogram 2.5 years before the current admission, and the chief complaint of the current admission was not chest pain, typical for Takotsubo cardiomyopathy, there is a possibility that dilated cardiomyopathy diagnosed 2.5 years before the current admission might have been attributed to the cardiac remodeling by chronic and recurrent Takotsubo cardiomyopathy.

It has been demonstrated that contractile reserve assessed by the administration of catecholamine predicts long-term prognosis in patients with dilated cardiomyopathy [16–18]. Therefore, whatever the etiology of cardiac dysfunction of our patient is, improved left basal contraction during the advent of Takotsubo cardiomyopathy, a potential *intrinsic* catecholamine-mediated cardiomyopathy, might indicate the fair prognosis of our patient, although circulating catecholamine levels are not always increased in Takotsubo cardiomyopathy [19].

## Conclusion

We showed a case who had been diagnosed with dilated cardiomyopathy who demonstrated left ventricular basal hypercontraction at the advent of Takotsubo cardiomyopathy on the latest admission. Such findings might provide important information on the possibility of chronic or recurrent Takotsubo cardiomyopathy as the underlying cause of dilated cardiomyopathy in some patients.
